# Arachidonic acid-derived hydroxyeicosatetraenoic acids are positively associated with colon polyps in adult males: a cross-sectional study

**DOI:** 10.1038/s41598-019-48381-0

**Published:** 2019-08-19

**Authors:** C. Austin Pickens, Zhe Yin, Lorraine M. Sordillo, Jenifer I. Fenton

**Affiliations:** 1 Department of Food Science and Human Nutrition, Michigan State University, East Lansing, MI USA; 2 College of Veterinary Medicine, Michigan State University, East Lansing, MI USA

**Keywords:** Predictive markers, Mass spectrometry, Colon cancer

## Abstract

Oxylipids are potent lipid mediators associated with inflammation-induced colon carcinomas and colon tumor survival. Therefore, oxylipid profiles may be useful as novel biomarkers of colon polyp presence. The aim of this study was to investigate the relationship between plasma non-esterified oxylipids and the presence of colon polyps. A total of 123 Caucasian men, ages 48 to 65, were categorized into three groups: those with no polyps, those with one or more hyperplastic polyps, and those with one or more adenomas. Plasma non-esterified oxylipids were analyzed using solid phase extraction and quantified using a targeted HPLC tandem mass spectrometric analysis. Statistical analyses included Kruskal-Wallis one-way ANOVA with Dunn’s test for multiple comparison and generalized linear models to adjust for confounding factors such as age, anthropometrics, and smoking status. In general, monohydroxy omega-6-derived oxylipids were significantly increased in those with polyps. Concentrations of 5-hydroxyeicosatetraenoic acid (HETE) and 11-HETE were significantly higher in those with hyperplastic polyps and adenomas compared to those with no polyps. Arachidonic acid-derived HETEs were significantly associated with colon polyp types, even after adjusting for age, smoking, and body mass index or waist circumference in regression models. Since many of these oxylipids are formed through oxygenation by lipoxygenases (i.e., 5-, 12-, and 15-HETE, and 15- hydroxyeicosatrienoic acid [HETrE]) or auto-oxidative reactions (i.e., 11-HETE), this may indicate that lipoxygenase activity and lipid peroxidation are increased in those with colon polyps. In addition, since oxylipids such as 5-, 12-, and 15-HETE are signaling molecules involved in inflammation regulation, these oxylipids may have important functions in inflammation-associated polyp presence. Future studies should be performed in a larger cohorts to investigate if these oxylipids are useful as potential biomarkers of colon polyps.

## Introduction

Colon polyps are formed by abnormal growth of tissue on the lining of the colon^1^. Polyps can be categorized based on number, size, and histologic characteristics, such as hyperplastic polyps or adenomas. Intestinal polyps can be classified based on polyp type as non-neoplastic or neoplastic. Hyperplastic polyps are benign epithelial proliferations that are classified as non-neoplastic polyps. Adenomas are the most common type of neoplastic polyp that can progress toward colorectal cancer. Currently, colon polyps are identified during colonoscopy screening, and although most polyps do not become cancerous, polyp removal decreases future colon cancer risk^1,2^. Since age is a risk factor associated with colorectal cancer, it is recommended that all adults in the United States have regular examinations by age 50. However, recent epidemiological studies report an increased incidence of colorectal cancer in the United States, even in young adults under the recommended screening age^3,4^. Colon polyp risk factors include age (>50 years), sex, obesity, and lifestyle (lack of physical activity, alcohol consumption, and tobacco use). Higher body mass index (BMI) is associated with elevated risk of colorectal adenomas^5^, and more recently Comstock *et al*. also reported that obese men are more likely to have adenomas^6^.

Pathology associated with obesity contributes to chronic low-grade inflammation which plays a role in the development and pathogenesis of colon polyps^1^. In obesity, chronic low-grade inflammation is associated with excess lipid accumulation in white adipose tissue (WAT) which triggers monocyte recruitment and activation of M1 macrophages^7,8^. In fact, macrophages are major sources of oxidized lipid mediators deemed oxylipids^9^, which are potent bioactive lipid mediators endogenously synthesized. Oxylipids can be synthesized enzymatically by cytochrome P450 (CYP) enzymes^10^, cyclooxygenases (COX)^11^, and lipoxygenase (LOX)^12^. However, oxylipids are also produced through non-enzymatic autoxidation mechanisms (i.e., free radicals)^9^. Omega-3 (n-3) and omega-6 (n-6) polyunsaturated fatty acids (PUFAs) are structurally similar oxylipid substrates, and accumulation of one PUFA family can lead to enzymatic competition and alter downstream lipid metabolism. Most n-3 PUFA metabolites are considered to be anti-inflammatory while those from n-6 PUFA are considered to be pro-inflammatory in cancer models^9^. Oxylipid function is dependent on the oxidation pathway and the precursor PUFA substrate, and oxylipid signaling mechanisms include angiogenesis, cell growth, and either activating/enhancing or resolving inflammatory responses^13^.

Western diets are typically high in n-6 PUFAs such as linoleic acid (LA), which can alter the ratio of PUFAs leading to a shift towards n-6 fatty acid elongation and desaturation, resulting in accumulation of arachidonic acid (ARA) and ARA-derived oxylipids. It is suspected that altered lipid metabolism can play a role in the transformation of colorectal polyps to colorectal cancer^14–16^. Previous studies investigated PUFA dietary intake effects on adenomas and colon cancer risk, however, the results from these studies are mixed^17–19^. Dietary recalls and nutritional assessments are subject to bias from participants, thus, investigating PUFA biochemical markers and metabolites may identify biomarkers associated with colon polyp risk. In our study, 63 plasma oxylipids were quantified to determine oxylipid classes and concentrations significantly associated with colon polyp presence and type in 123 males. After false discovery correction and across all statistical analyses, the plasma ARA-derived oxylipids 5- and 11-hydroxyeciosatetreanoic acid (HETEs) were significantly elevated and associated with polyp type. The effects remained significant even after normalizing these HETEs to ARA concentrations and adjusting for confounding factors such as age, smoking, body mass index (BMI), and waist circumference (WC). In addition, concentration ranges of these HETEs were associated with an increased likelihood of polyp presence.

## Methods and Materials

### Ethics statement

The study was approved by the Biomedical and Health Institutional Review Board (IRB) of Michigan State University (IRB# 08-786). All methods were performed in accordance with the relevant guidelines and regulations of the Michigan State University IRB. At the time of enrollment, immediately prior to routine colonoscopy, written informed consent was obtained from each participant.

### Study population and polyp identification

A group of asymptomatic and >96% Caucasian males ranging from 48–65 years of age were recruited between August 2009 and February 2011 in a cross-sectional study as previously described^6–8,20–24^. Exclusion criteria included but not limited to: 1) cancer in the past two years, 2) diabetes, 3) autoimmune diseases, etc. A detailed list of exclusion criteria can be found in the previous published paper^21^. Clinical metadata on participants’ co-morbidities, medication status, and family history were obtained at the time of enrollment immediately prior to full colonoscopy. During the colonoscopy, a gastroenterologist (MSU-affiliated clinics, MI) categorized each polyp based on the location and board-certified pathologists assigned a polyp type to each specimen collected. The participants were grouped based on their polyp types as: 1) those with no polyp, 2) those with one or more hyperplastic polyps, and 3) those with one or more adenomas. Anthropometric measures (i.e. height, weight, and waist circumference) of study subjects were taken by trained medical professionals and these anthropometrics measures were used to calculate BMI. Venous blood of participants was drawn and plasma fraction was isolated by centrifugation and stored at −80 °C until analysis. Smoking status was assigned to subjects as “ever smoked” or “never smoked”. A complete description of the study design can be found elsewhere^6^.

### Non-esterified plasma PUFA and oxylipid extraction, isolation and analysis

Non-esterified plasma PUFA and oxylipid extraction with internal standards and isolation were carried out as previously reported by our group and are described in detail^20,25^. In brief, 500 μL of each patient’s plasma was used for lipid extraction. Deuterium labeled internal standards were added. Phenomenex Strata-X (60 mg/3 mL, Phenomenex, Torrane, CA) was used to perform solid-phase extraction for each sample as previously described^7,20^. The prepared samples were stored under high-purity argon at −20 °C for no longer than a week. Liquid chromatography tandem mass spectrometry (LC/MS/MS) analysis was conducted as previously reported^20^. In brief, liquid chromatography separations were carried out on a Waters ACQUITY UPLC system (Waters, Milford, MA) using an Ascentis Express C18 column (10 cm × 2.1 mm; 2.7 μm particles, Sigma-Aldrich, St. Louis, MO) which was connected to a Waters Xevo TQ-S triple quadrupole mass spectrometer (Waters, Milford, MA). Peak detection, integration, and quantification were performed using TargetLynx (Waters) software.

### Data merging and oxylipid exclusion

Sixty-three PUFAs and oxylipids were quantified by LC/MS/MS analyses. Analytes with >50% missing values were excluded and 34 remaining analytes in the data matrix were: LA and LA-derived 9-hydroxyoctadecadienoic acid (HODE), 13-HODE, 9-oxooctadecadienoic acid (KODE), 13-KODE, 9,10-EpOME, 12,13-EpOME, 9,10-dihydroxyoctadecadienoic acid (DiHOME) and 12,13-DiHOME, alpha-linolenic acid(ALA)-derived 13-hydroxyoctadecatrienoic acid (HOTrE), dihomo-gamma linolenic acid (DGLA)-derived 15-hydroxyeicosatrienoic acid (HETrE), ARA and ARA-derived 5-HETE, 11-HETE, 12-HETE, 15-HETE, 20-HETE, 14,15-epoxyeicosatrienoic acid (EET), 8,9-dihydroxyeicosatrienoic acid (DHET), 11,12-DHET, 14,15-DHET, arachidonoyl ethanolamide (AEA), arachidonoyl glycerol (AG), leukotriene B4 (LTB4), prostaglandin E2 (PGE2), thromboxane B2 (TBX2), EPA and EPA-derived 5,6-dihydroxyeicosatetraenoic acid (DiHETE), 17,18-DiHETE, DHA and DHA-derived 19,20-dihydroxydocosapentaenoic acid (HDPA), docosahexaenoyl ethanolamide (DHEA), and Sphingosine-1-phosphate (S1P). A comprehensive list metabolites, quantitative selected reaction monitoring transitions and labeled internal standards used in our study were previously reported^20^.

### Statistical analyses

The median, Q1, and Q3 were calculated for individuals’ characteristic variables (Table 1). The quantiles of concentrations of each plasma PUFA and oxylipid are also presented in Table 1. Plasma non-esterified LA, 12,13-DiHOME, and ARA values are presented as fold changes, since a significant proportion of individual’s concentrations were above the highest standard curve and re-extraction or reanalysis were not possible due to limited quantities of plasma. The internal standards utilized for 12,13-DiHOME was 8,9-DHET-*d*11 (Cayman Chemical, Ann Arbor, MI), and LA and ARA utilized ARA-*d*8 (Cayman Chemical) as an internal standard. Oxylipid totals were calculated as follows: Total HODE = ∑ 9-HODE + 13-HODE, Total KODE = ∑ 9-KODE + 13-KODE, Total EpOME = ∑ 9,10-EpOME + 12,13-EpOME, Total DiHOME = ∑ 9,10-DiHOME 12,13-DiHOME, Total HETE = ∑ 5-HETE + 11-HETE + 12-HETE + 15-HETE + 20-HETE, Total DHET = ∑ 8,9-DHET + 11,12-DHET + 14,15-DHET, Total DiHETE = ∑ 5,6-DiHETE + 17,18-DiHETE. Missing PUFA and oxylipid values were imputed as square root of each respective minimum value in all statistical analyses as previously described^20^.

The participants were grouped based on the polyp types as previously mentioned^20,25^. Statistical differences in age, BMI, WC, and non-esterified PUFA and oxylipids concentrations among three groups of participants were determined by Kruskal Wallis one-way analysis of variance (ANOVA) and Dunn’s test for multiple comparison. P-values were corrected for false discoveries according to Benjamini-Hochberg (BH) and Bonferroni (Bon)^26,27^. Polyp types (i.e., hyperplastic and adenoma) were regressed on the concentrations of oxylipids and clinical covariates, including age and smoking status of participants, using generalized linear model assuming a Poisson distribution. Model 1 included log-transformed explanatory variables (PUFA, oxylipids, or oxylipid product-to precursor ratios) and was adjusted for age and smoking status. Since BMI and WC are highly correlated, two additional models were run adjusting for age and smoking along with either BMI or WC (Table 2). Next, each individual oxylipid concentration was normalized to its precursor PUFA to determine whether differences in oxylipid concentrations were due to altered PUFA availability or metabolism (Table 3). Finally, simple logistic regression models, adjusted for age, smoking, and BMI, were used to calculate polyp presence likelihood calculate across oxylipids concentrations categorized into tertiles (Table 4). Test for exposure was conducted to determine if increases in oxylipid tertiles compared to the lowest tertile were associated with having polyps. The test for trend was conducted to determine if increase in oxylipid tertiles were associated with polyps compared to individuals without polyps. All the statistical analyses, except logistic regressions, were performed using R v3.2.2^28^. Logistic regressions were performed using SAS version 9.4 (SAS, Cary, NC).

## Results

### Characteristics and oxylipid concentrations of study populations

Age, BMI, WC, smoking status, and non-esterified PUFA and plasma oxylipid concentrations for the overall population (n = 123) are separated into three groups based on the polyp type (Table 1). Of the 123 participants, 67 (54.5%) had no polyps, 20 (16.3%) had hyperplastic polyps, and 36 (29.3%) had adenomas. Non-parametric ANOVAs were carried out for each variable across these three groups. Age did not differ across the three groups. BMI was significantly higher in participants with adenomas. Participants with hyperplastic polyps or adenomas had increased WCs compared to those with no polyps. LA-derived oxylipid concentrations did not differ across the different polyp types. The levels of 15-HETrE, which is a DGLA-derived oxylipid, were higher in individuals with adenomas compared to no polyps. Concentrations of ARA-derived 5-, 11-, and 15-HETE were all increased significantly in participants with hyperplastic polyps and adenomas compared to those with no polyps.

**Table 1:** Presence of polyps is associated with increased plasma concentrations of monohydroxy arachidonic acid-derived oxylipids (values expressed as median [Q1, Q3]).

Variable	Overall	No polyp	Hyperplastic	Adenoma	BH FDR p-value^c^
Sample size (n = )	123	67	20	36	
Ever Smoked (% total)	30	27	29	39	
Age (years)	58.0 [53.0, 60.5]	57.0 [53.0, 61.0]^A^	57.5 [52.8, 60.0]^A^	58.0 [53.0, 60.3]^A^	—
BMI (kg/m^2^)	29.3 [26.1, 32.7]	28.2 [24.6, 30.9]^A^	28.3 [26.0, 31.8]^A^	32.2 [28.9, 35.9]^B^	p ≤ 0.05
WC (in)	41.5 [37.0, 45.3]	40.0 [35.9, 44.3]^A^	41.0 [38.0, 44.4]^AB^	43.8 [40.0, 48.6]^B^	*
Oxylipids (nM)					—
LA^a^	0.7 [0.4, 0.9]	0.7 [0.4, 1.1]^A^	0.7 [0.5, 1.0]^A^	0.7 [0.4, 0.8]^A^	—
9-HODE	14.3 [9.4, 23.9]	15.8 [8.8, 27.4]^A^	16.1 [12.4, 21.9]^A^	13.5 [10.6, 22.8]^A^	—
13-HODE	13.4 [8.6, 22.0]	13.4 [8.4, 24.1]^A^	14.5 [9.6, 18.8]^A^	12 [9.3, 19.5]^A^	—
Total HODE	29.3 [19.1, 47.4]	30.0 [17.3, 51.4]^A^	30.2 [22.1, 40.1]^A^	23.6 [20.1, 45.7]^A^	—
9-KODE	7.9 [5.1, 12.01]	7.7 [4.9, 12.8]^A^	9.8 [5.4, 11.8]^A^	7.7 [5.1, 11.4]^A^	—
13-KODE	2.2 [1.4, 3.8]	2.1 [1.4, 3.7]^A^	2.8 [1.8, 4.3]^A^	2.2 [1.5, 3.1]^A^	—
Total KODE	11.0 [6.7,15.7]	11.6 [6.5, 18.0]^A^	13.0 [8.5, 15.7]^A^	10.4 [7.0, 14.6]^A^	—
9,10-EpOME	21.6 [15.0, 32.7]	19.8 [14.4, 33.9]^A^	22.8 [18.0, 41.3]^A^	20.4 [15.5, 29.0]^A^	—
12,13-EpOME	40.2 [27.0, 57.9]	45.6 [27.9, 60.3]^A^	42.6 [26.9, 60.8]^A^	38.7 [25.5, 52.5]^A^	—
Total EpOME	64.2 [42.6, 94.2]	58.8 [42.6, 97.5]^A^	68.1 [36.6, 104.1]^A^	62.1 [44.4, 75.3]^A^	—
9,10-DiHOME	8.4 [4.7,16.4]	8.7 [5.0, 17.6]^A^	8.0 [3.9, 14.0]^A^	8.1 [4.7, 14.3]^A^	—
12,13-DiHOME^a^	3.3 [2.1, 5.7]	3.6 [2.2, 5.9]^A^	3.2 [2.0, 4.9]^A^	3.2 [2.0, 6.5]^A^	—
Total DiHOME	12.0 [7.1, 23.2]	12.6 [7.5, 23.2]^A^	13.0 [5.8, 20.3]^A^	11.4 [7.1, 22.0]^A^	—
13-HOTrE	2.0 [1.1, 3.9]	1.9 [1.3, 3.5]^A^	2.8 [1.7, 3.4]^A^	1.9 [1.1, 4.0]^A^	—
15-HETrE	0.4 [0.2, 0.6]	0.3 [0.2, 0.5]^A^	0.4 [0.3, 0.6]^AB^	0.5 [0.3, 0.6]^B^	0.066
ARA^a^	14.9 [10.4, 21.4]	14.6 [10.4, 20.8]^A^	15.4 [12.0, 21.4]^A^	15.2 [10.4, 21.9]^A^	—
5-HETE	2.8 [1.6, 9.5]	1.9 [1.2, 5.0]^A^	4.9 [3.3, 13.4]^B^	4.6 [2.4, 12.4]^B^	p ≤ 0.001
11-HETE	0.7 [0.3, 1.3]	0.4 [0.3, 0.8]^A^	0.9 [0.6, 1.5]^B^	0.8 [0.6, 1.6]^B^	p ≤ 0.005
12-HETE	9.4 [6.0, 15.3]	8.4 [5.0, 14.7]^A^	12.1 [7.8, 19.5]^A^	10.5 [6.5, 16.4]^A^	—
15-HETE	0.8 [0.5, 1.2]	0.7 [0.5, 1.1]^A^	1.1 [0.7, 1.4]^B^	1.0 [0.6, 1.5]^B^	*
20-HETE	0.8 [0.5, 1.2]	0.7 [0.5, 1.1]^A^	0.9 [0.4, 1.2]^A^	0.7 [0.5, 1.1]^A^	—
Total HETE	19.0 [10.9, 32.8]	13.8 [9.5, 21.1]^A^	22.9 [17.9, 34.0]^B^	21.2 [12.8, 41.0]^B^	p ≤ 0.05
14,15-EET	1.3 [1, 2.2]	1.3 [1.0, 2.1]^A^	1.2 [0.7, 2.2]^A^	1.9 [1.2, 2.3]^A^	—
8,9-DHET	0.6 [0.4, 0.8]	0.5 [0.4, 0.8]^A^	0.7 [0.5, 0.9]^A^	0.5 [0.4, 0.8]^A^	—
11,12-DHET	1.0 [0.8,1.4]	1.0 [0.7, 1.3]^A^	1.3 [0.8, 1.9]^A^	1.1 [0.8, 1.4]^A^	—
14,15-DHET	1.6 [1.3, 2.0]	1.5 [1.2, 2.0]^A^	1.8 [1.3, 2.8]^A^	1.6 [1.3, 1.9]^A^	—
Total DHET	3.2 [2.5, 4.2]	3.1 [2.4, 3.9]^A^	3.9 [2.8, 5.9]^A^	3.5 [2.6, 4.0]^A^	—
AEA	0.2 [0.2, 0.24]	0.2 [0.2, 0.2]^A^	0.2 [0.2, 0.3]^A^	0.2 [0.1, 0.2]^AB^	*
2-AG	1.4 [0.8, 1.99]	1.4 [0.8, 2.1]^AB^	1.8 [1.1, 2.1]^B^	1.2 [0.8, 1.7]^A^	*
LTB4	0.3 [0.2, 0.45]	0.3 [0.2, 0.4]^A^	0.3 [0.2, 0.4]^A^	0.3 [0.2, 0.8]^A^	—
PGE2	4.4 [2.2, 10.3]	5.5 [3, 10.4]^A^	6.0 [2.0, 21.7]^A^	3.4 [1.7, 6.5]^A^	—
TXB2	0.3 [0.2, 0.9]	0.3 [0.2, 0.7]^A^	0.4 [0.2, 1.2]^A^	0.4 [0.2, 0.8]^A^	—
EPA^b^	0.2 [0.1, 0.3]	0.2 [0.1, 0.4]^A^	0.2 [0.1,0.3]^A^	0.2 [0.1, 0.3]^A^	—
5,6-DiHETE	5.0 [3.0, 7.3]	5.1 [2.9, 6.5]^A^	5.0 [3.3, 6.7]^A^	4.9 [3.0, 8.0]^A^	—
17,18-DiHETE	8.5 [5.9, 14.3]	8.6 [6.2, 15.3]^A^	8.3 [5.9, 14.1]^A^	8.1 [4.8, 10.4]^A^	—
Total DiHETE	13.4 [9.5, 25.0]	12.8 [9.3, 24.8]^A^	14.2 [10.1, 24.7]^A^	15.0 [10.4, 28.5]^A^	—
DHA^b^	4.6 [3.3,7.8]	5.6 [3.6,8.5]^A^	4.5 [3.5, 7.9]^A^	4.2 [3.1, 6.3]^A^	—
19,20-HDPA	2.8 [2.1,4.42]	2.9 [2.2, 4.5]^A^	2.7 [2.4, 4.2]^A^	3.0 [1.7, 3.8]^A^	—
DHEA	0.5 [0.3, 0.8]	0.6 [0.4, 0.7]^A^	0.6 [0.3, 0.8]^A^	0.5 [0.2, 0.9]^A^	—
S1P	37.7 [26.7, 47.1]	35.9 [25.4, 45.7]^A^	39.3 [25.6, 51.1]^A^	39.8 [31.5, 51.6]^A^	—
9-KOHO	0.5 [0.4, 0.7]	0.5 [0.4, 0.6]^A^	0.6 [0.4, 0.8]^A^	0.5 [0.4, 0.7]^A^	—
13-KOHO	0.2 [0.1, 0.2]	0.2 [0.1, 0.2]^A^	0.2 [0.1, 0.3]^A^	0.2 [0.1, 0.2]^A^	—
9,10-DiEp	0.4 [0.2, 0.9]	0.4 [0.2, 1.1]^A^	0.3 [0.1, 0.7]^A^	0.4 [0.2, 0.7]^A^	—
12,13-DiEp	0.1 [0.1, 0.1]	0.1 [0.1, 0.1]^A^	0.1 [0.1, 0.1]^A^	0.1 [0.1, 0.1]^A^	—
14,15-DHEp	1.3 [0.9, 1.8]	1.3 [0.9, 1.8]^A^	1.8 [1.3, 3.2]^A^	1.0 [0.8, 1.3]^A^	—

Oxylipid totals were calculated as follows: Total HODE calculated as ∑ 9-HODE + 13-HODE; Total KODE calculated as ∑ 9-KODE + 13-KODE; Total EpOME calculated as ∑ 9,10-EpOME + 12,13-EpOME; Total DiHOME calculated as ∑ 9,10-DiHOME 12,13-DiHOME; Total HETE calculated as ∑ 5-HETE + 11-HETE + 12-HETE + 15-HETE + 20-HETE; Total DHET calculated as ∑ 8,9-DHET + 11,12-DHET + 14,15-DHET; Total DiHETE calculated as ∑ 5,6-DiHETE + 17,18-DiHETE.

^a^Plasma non-esterified LA, 12,13-DiHOME, and AA values are presented as levels (i.e., peak area of compound/ peak area of internal standard), since a majority of participant’s concentrations were greater (i.e., > 10 fold) than the highest standard curve value.

^b^Plasma non-esterified EPA and DHA are expressed as μM.

^c^Categorical analysis of polyp severity by Kruskal Wallis one-way ANOVA and Dunn’s test for multiple comparison. P-values were corrected for false discovery rate (FDR) according to Benjamini-Hochberg (BH).

### Non-esterified plasma oxylipids are associated with polyp type

**Figure 1:**
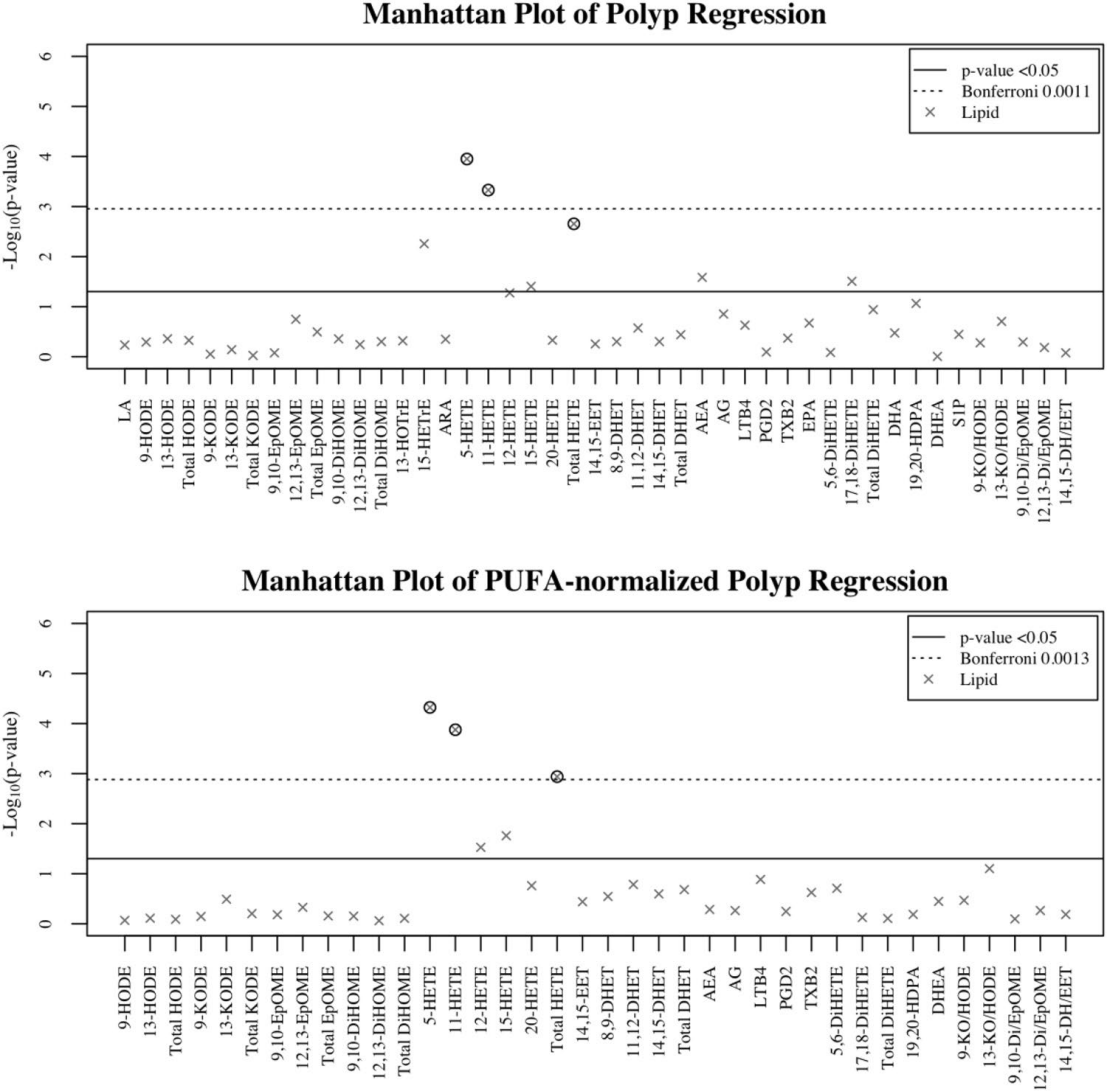
Manhattan plots of polyp type regressed on non-esterified plasma polyunsaturated fatty acids, oxylipids, and PUFA-normalized oxylipids. Manhattan plots of the −log10(p-value) for polyp type regressed on log transformed PUFAs, oxylipids, and oxylipid parent-to-precursor ratios individually (i.e., one at a time). Single non-esterified plasma fatty acid, oxylipid, and product to precursor ratio regression models are defined as: polyp type = age + smoking + log(oxylipid) and polyp type = age + smoking + log(oxylipid/PUFA). Each y-axis represents −log10(p-value) for each respective model, and x-axis represent the plasma non-esterified fatty acid, oxylipid, and oxylipid product-to-precursor ratios. Plasma lipids with Benjamini-Hochberg false discovery rate p-values ≤ 0.05 are circled. Oxylipids totals calculated as follows: Total HODE = ∑ 9-HODE + 13-HODE; Total KODE = ∑ 9-KODE + 13-KODE; Total EpOME = ∑ 9,10-EpOME + 12,13-EpOME; Total HETE = ∑ 5-HETE + 11-HETE + 12-HETE + 15-HETE + 20-HETE; Total DHET = ∑ 8,9-DHET + 11,12-DHET + 14,15-DHET; Total DiHETE = ∑ 5,6-DiHETE + 17,18-DiHETE; 9-KO/HODE = 9-KODE/9-HODE; 13-KO/HODE = 13-KODE/13-HODE; 9,10-Di/EpOME = 9,10-DiHOME/9,10-EpOME; 12,13-Di/EpOME = 12,13-DiHOME/12,13-EpOME; 14,15-DH/EET = 14,15-DHET/14,15-EET.

Generalized linear models were used to estimate the effects of each PUFA and oxylipid associated with polyp type. Poisson distribution was assumed for polyp type due to the distribution of patients in each group (Table 1), and models were adjusted for age and smoking status. In all regression models, the response variable polyp type was regressed on concentrations of oxylipids and their precursor PUFAs (Table 2 and Fig. 1 top), or PUFA-normalized oxylipid concentrations (Table 3 and Fig. 1 bottom). The −log10(p-values) from these single lipid regressions are presented as a Manhattan plot (Fig. 1), and p-values that were significant after BH FDR adjustment were circled, and p-values significant below the Bonferroni cutoff are displayed above the dashed line. 15-HETrE derived from DGLA, 5-, 11-, 15-HETEs and AEA derived from ARA, and 17,18-DiHETE derived from EPA were significantly associated with polyp type. 5-HETE and 11-HETE remained significant even after FDR p-value adjustment.

**Table 2:** Association of arachidonic acid-derived oxylipids are associated with colon polyp type.

Oxylipids	Age + Smoking				Age + Smoking + BMI				Age + Smoking + WC			
	Beta	p-value	BH^a^ p-value	Bon^a^ p-value	Beta	p-value	BH^a^ p-value	Bon^a^ p-value	Beta	p-value	BH^a^ p-value	Bon^a^ p-value
LA	−0.10	—	—	—	−0.11	—	—	—	−0.09	—	—	—
9-HODE	−0.10	—	—	—	−0.05	—	—	—	−0.06	—	—	—
13-HODE	−0.12	—	—	—	−0.04	—	—	—	−0.05	—	—	—
Total HODE	−0.11	—	—	—	−0.04	—	—	—	−0.05	—	—	—
9-KODE	−0.02	—	—	—	0.06	—	—	—	0.05	—	—	—
13-KODE	0.05	—	—	—	−0.02	—	—	—	0.01	—	—	—
Total KODE	−0.01	—	—	—	0.04	—	—	—	0.04	—	—	—
9,10-EpOME	−0.04	—	—	—	−0.02	—	—	—	0.00	—	—	—
12,13-EpOME	−0.23	—	—	—	−0.07	—	—	—	−0.10	—	—	—
Total EpOME	−0.19	—	—	—	−0.05	—	—	—	−0.06	—	—	—
9,10-DiHOME	−0.08	—	—	—	0.06	—	—	—	0.00	—	—	—
12,13-DiHOME	−0.07	—	—	—	0.16	—	—	—	0.07	—	—	—
Total DiHOME	−0.08	—	—	—	0.10	—	—	—	0.02	—	—	—
13-HETrE	0.08	—	—	—	0.08	—	—	—	0.11	—	—	—
15-HETrE	0.34	p ≤ 0.01	0.062	—	0.25	0.051	—	—	0.28	p ≤ 0.05	—	—
AA	−0.13	—	—	—	−0.19	—	—	—	−0.16	—	—	—
5-HETE	0.34	p = 0.0001	p ≤ 0.01	p ≤ 0.01	0.28	p ≤ 0.005	—	—	0.30	p ≤ 0.005	0.067	0.067
11-HETE	0.34	p ≤ 0.0005	p ≤ 0.05	p ≤ 0.05	0.28	p ≤ 0.01	—	—	0.29	p ≤ 0.005	—	—
12-HETE	0.26	0.053	—	—	0.20	—	—	—	0.23	0.090	—	—
15-HETE	0.26	p ≤ 0.05	—	—	0.17	—	—	—	0.20	—	—	—
20-HETE	0.13	—	—	—	−0.04	—	—	—	0.04	—	—	—
Total HETE	0.42	p ≤ 0.005	p ≤ 0.05	—	0.32	p ≤ 0.05	—	—	0.35	p ≤ 0.05	—	—
14,15-EET	0.09	—	—	—	0.00	—	—	—	0.01	—	—	—
8,9-DHET	0.15	—	—	—	0.18	—	—	—	0.17	—	—	—
11,12-DHET	0.27	—	—	—	0.36	—	—	—	0.35	—	—	—
14,15-DHET	0.19	—	—	—	0.26	—	—	—	0.29	—	—	—
Total DHET	0.25	—	—	—	0.33	—	—	—	0.33	—	—	—
AEA	−0.58	p ≤ 0.05	—	—	−0.63	p ≤ 0.05	—	—	−0.58	p ≤ 0.05	—	—
2-AG	−0.24	—	—	—	−0.20	—	—	—	−0.19	—	—	—
LTB4	0.16	—	—	—	0.12	—	—	—	0.12	—	—	—
PGE2	0.02	—	—	—	0.09	—	—	—	0.06	—	—	—
TXB2	0.09	—	—	—	0.08	—	—	—	0.10	—	—	—
EPA	−0.11	—	—	—	−0.09	—	—	—	−0.08	—	—	—
5,6-DiHETE	0.03	—	—	—	0.07	—	—	—	0.07	—	—	—
17,18-DiHETE	−0.27	p ≤ 0.05	—	—	−0.13	—	—	—	−0.15	—	—	—
Total DiHETE	−0.24	—	—	—	−0.08	—	—	—	−0.11	—	—	—
DHA	−0.15	—	—	—	−0.17	—	—	—	−0.13	—	—	—
19,20-HDPA	−0.33	0.086	—	—	−0.12	—	—	—	−0.14	—	—	—
DHEA	0.00	—	—	—	−0.01	—	—	—	0.03	—	—	—
S1P	0.22	—	—	—	0.11	—	—	—	0.20	—	—	—
9-KOHO	0.12	—	—	—	0.17	—	—	—	0.16	—	—	—
13-KOHO	0.23	—	—	—	0.03	—	—	—	0.10	—	—	—
9,10-DiEp	−0.07	—	—	—	0.06	—	—	—	0.00	—	—	—
12,13-DiEp	0.06	—	—	—	0.22	—	—	—	0.15	—	—	—
14,15-DHEp	−0.03	—	—	—	0.10	—	—	—	0.09	—	—	—

Beta coefficients and p-values were determined through general linear regression with Poisson distribution. Models were defined as Polyp type = Age + Smoking + log(oxylipid), Polyp type = Age + Smoking + BMI + log(oxylipid), and Polyp type = Age + Smoking + WC + log(oxylipid). P-values were adjusted for false discovery rate (FDR) according to Benjamini-Hochberg and Bonferroni. Only p-values < 0.1 are displayed. Oxylipids totals calculated as follows: Total HODE = Σ 9-HODE + 13-HODE; Total KODE = Σ 9-KODE + 13-KODE; Total EpOME = Σ 9,10-EpOME + 12,13-EpOME; Total HETE = Σ 5-HETE + 11-HETE + 12-HETE + 15-HETE + 20-HETE; Total DHET = Σ 8,9-DHET + 11,12-DHET + 14,15-DHET; Total DiHETE = Σ 5,6-DiHETE + 17,18-DiHETE.

^a^P-values were corrected for false discovery rate (FDR) according to Benjamini-Hochberg (BH) and Bonferroni (Bon).

PUFAs serve as substrates for oxylipids and are obtained through dietary intake. In order to assess whether differences in these oxylipids are due to altered levels of PUFA availability in the body or altered lipid metabolism, each oxylipid was normalized to its parent PUFA. Polyp type was then regressed on PUFA-normalized oxylipids with models adjusting for age and smoking status (Fig. 1 bottom and Table 3). ARA-derived 5-, 11-, 12-, and 15-HETE were positively associated with polyp type. Only 5- and 11-HETE remained significant after adjusting p-values for FDR. Results from non-parametric ANOVAs indicated BMI and WC were significantly different across polyp type. Therefore, two additional models were analyzed to adjust for BMI and WC separately to see if any lipids remained associated with polyp type. Model 1 adjusted for age and smoking, model 2 adjusted for age, smoking, and BMI, and model 3 adjusted for age, smoking, and WC (Tables 2 and 3). Across all three models, only ARA-derived 5- and 11-HETE remained significant after adjusting for age, smoking, and either BMI and WC (Table 2). After normalization to parent PUFA concentrations, 5-, and 11-HETE remained significant across all models even after FDR correction (Table 3).

**Table 3:** After PUFA-normalization arachidonic acid-derived oxylipids remain significantly associated with polyp type.

Oxylipids	Age + Smoking				Age + Smoking + BMI				Age + Smoking + WC			
	Beta	p-value	BH^a^ p-value	Bon^a^ p-value	Beta	p-value	BH p-value	Bon p-value	Beta	p-value	BH p-value	Bon p-value
9-HODE	−0.03	—	—	—	0.03	—	—	—	0.01	—	—	—
13-HODE	−0.04	—	—	—	0.03	—	—	—	0.01	—	—	—
Total HODE	−0.03	—	—	—	0.03	—	—	—	0.01	—	—	—
9-KODE	0.06	—	—	—	0.19	—	—	—	0.15	—	—	—
13-KODE	0.18	—	—	—	0.08	—	—	—	0.12	—	—	—
Total KODE	0.09	—	—	—	0.18	—	—	—	0.16	—	—	—
9,10-EpOME	0.10	—	—	—	0.14	—	—	—	0.14	—	—	—
12,13-EpOME	−0.11	—	—	—	0.04	—	—	—	0.00	—	—	—
Total EpOME	−0.07	—	—	—	0.07	—	—	—	0.04	—	—	—
9,10-DiHOME	−0.03	—	—	—	0.08	—	—	—	0.02	—	—	—
12,13-DiHOME	−0.02	—	—	—	0.15	—	—	—	0.08	—	—	—
Total DiHOME	−0.03	—	—	—	0.10	—	—	—	0.04	—	—	—
5-HETE	0.36	p ≤ 0.0001	p ≤ 0.005	p ≤ 0.005	0.31	p ≤ 0.001	p ≤ 0.05	p ≤ 0.05	0.32	p ≤ 0.0005	p ≤ 0.05	p ≤ 0.05
11-HETE	0.38	p ≤ 0.0005	p ≤ 0.005	p ≤ 0.01	0.34	p ≤ 0.005	p ≤ 0.05	p ≤ 0.05	0.35	p ≤ 0.001	p ≤ 0.05	p ≤ 0.05
12-HETE	0.25	p ≤ 0.05	—	—	0.23	p ≤ 0.05	—	—	0.24	p ≤ 0.05	—	—
15-HETE	0.28	p ≤ 0.05	—	—	0.23	0.059	—	—	0.24	p ≤ 0.05	—	—
20-HETE	0.22	—	—	—	0.13	—	—	—	0.16	—	—	—
Total HETE	0.40	p ≤ 0.005	p ≤ 0.05	p ≤ 0.05	0.35	p ≤ 0.005	0.062	—	0.36	p ≤ 0.005	0.054	—
14,15-EET	0.10	—	—	—	0.08	—	—	—	0.07	—	—	—
8,9-DHET	0.16	—	—	—	0.22	—	—	—	0.18	—	—	—
11,12-DHET	0.22	—	—	—	0.30	0.064	—	—	0.27	—	—	—
14,15-DHET	0.19	—	—	—	0.27	—	—	—	0.25	—	—	—
Total DHET	0.21	—	—	—	0.29	—	—	—	0.26	—	—	—
AEA	−0.11	—	—	—	−0.09	—	—	—	−0.10	—	—	—
2-AG	−0.08	—	—	—	−0.03	—	—	—	−0.04	—	—	—
LTB4	0.17	—	—	—	0.16	—	—	—	0.15	—	—	—
PGE2	0.05	—	—	—	0.11	—	—	—	0.08	—	—	—
TXB2	0.12	—	—	—	0.14	—	—	—	0.14	—	—	—
5,6-DiHETE	0.10	—	—	—	0.11	—	—	—	0.10	—	—	—
17,18-DiHETE	−0.03	—	—	—	0.03	—	—	—	0.02	—	—	—
Total DiHETE	0.03	—	—	—	0.07	—	—	—	0.05	—	—	—
19,20-HDPA	−0.07	—	—	—	0.09	—	—	—	0.06	—	—	—
DHEA	0.14	—	—	—	0.15	—	—	—	0.15	—	—	—
9-KOHO	0.14	—	—	—	0.19	—	—	—	0.17	—	—	—
13-KOHO	0.30	0.079	—	—	0.12	—	—	—	0.17	—	—	—
9,10-DiEp	−0.02	—	—	—	0.06	—	—	—	0.02	—	—	—
12,13-DiEp	0.06	—	—	—	0.14	—	—	—	0.10	—	—	—
14,15-DHEp	0.06	—	—	—	0.20	—	—	—	0.17	—	—	—

Beta coefficients and p-values were determined through general linear regression with Poisson distribution. Models were defined as Polyp type = Age + Smoking + log(oxylipid/PUFA), Polyp type = Age + Smoking + BMI + log(oxylipid/PUFA), and Polyp type = Age + Smoking + WC + log(oxylipid/PUFA). P-values were adjusted for false discovery rate (FDR) according to Benjamini-Hochberg and Bonferroni. Only p-values < 0.1 are displayed. Oxylipids totals calculated as follows: Total HODE = Σ 9-HODE + 13-HODE; Total KODE = Σ 9-KODE + 13-KODE; Total EpOME = Σ 9,10-EpOME + 12,13-EpOME; Total HETE = Σ 5-HETE + 11-HETE + 12-HETE + 15-HETE + 20-HETE; Total DHET = Σ 8,9-DHET + 11,12-DHET + 14,15-DHET; Total DiHETE = Σ 5,6-DiHETE + 17,18-DiHETE.

**Table 4:** Non-esterified plasma oxylipids concentrations of 5- and 11-HETE, as tertiles, are positively associated with polyp presence.

Plasma Concentration^a^	Test for exposure	Test for trend
	OR [95% CI]^b^	OR (p trend)^c^
5-HETE (nM)		**2**.**32**
≤1.86	1	**(p ≤ 0**.**005)**
>1.86 to ≤5.01	**5**.**14 [1**.**74**, **15**.**24]**	
>5.01	**6**.**13 [2**.**07**, **18**.**42]**	
11-HETE (nM)		**2**.**31**
≤0.39	1	**(p ≤ 0**.**005)**
>0.39 to ≤0.89	**3**.**88 [1**.**36**, **11**.**08]**	
>0.89	**5**.**72 [1**.**94**, **16**.**88]**	

^a^Plasma non-esterified oxylipids were separated into tertiles.

^b^Test for exposure was conducted to determine if increases in oxylipid tertiles, compared to the lowest tertile, were associated with having polyps. Odds ratio (OR) [95% Confidence Interval (CI)] are displayed.

^c^Test for trend was conducted to determine if increases in oxylipid tertiles were associated with presence of polyps compared to no polyps. Odds ratio (p-value) are displayed.

^b,c^Polytomous logistic regression was used to regress polyp presence on oxylipid tertiles. All data is referenced against the no polyps’ category. Both test for trend and test for exposure were adjusted for body mass index, age, and smoking. P-values bolded if p ≤ 0.05.

### Presence of colon polyps is associated with specific concentrations of oxylipids

Next, odds ratios (OR) were determined using polytomous logistic regressions to determine concentration ranges of 5- and 11-HETE that were associated with presence of 1 or more colon polyps compared to individuals with no colon polyps. The concentrations 5- and 11-HETE were separated into tertiles to provide the specific ranges that were most likely to be associated with the presence of polyps. In all models, polyp presence was regressed on oxylipid tertiles and all data is referenced against the no polyps’ group. Models were adjusted for age, smoking, and BMI, and odds ratios and confidence intervals were calculated. Individuals with >1.86 nM 5-HETE were five to six times more likely to have polyps than individuals with ≤ 1.86 nM 5-HETE. Results for 11-HETE were similar to 5-HETE, participants with 11-HETE concentrations >0.39 nM were three to five times more likely to have polyps than individuals with ≤ 0.39 nM 11-HETE. Overall, Individuals with polyps were two times more likely to have a higher concentration of 5- and 11-HETE compared to individuals without polyps.

## Discussion

The purpose of this study was to identify PUFA substrates and oxylipids associated with presence of colon polyps. We employed LC-MS/MS to analyze plasma PUFAs and oxylipids of participants. We report associations between higher concentrations of pro-inflammatory oxylipids and the presence of colon polyps. Specifically, we report elevated concentrations of ARA-derived 5-HETE and 11-HETE were associated with an increased likelihood of polyp presence. Associations between 5- and 11-HETE concentrations with polyp type remained significant after normalization to parent PUFA (i.e., ARA) concentrations, accounting for age, smoking, and either BMI or WC, and adjusting p-values for false discoveries. However, non-esterified plasma PUFAs were not associated with polyp in our study. Our results suggest that presence of colon polyps is associated with oxylipids that are metabolized through certain pathways, not entirely due to altered dietary PUFA intake^20^.

Cyclooxygenases and lipoxygenases are PUFA oxygenating enzymes. ARA-derived oxylipids produced through COX or LOX pathways are often pro-inflammatory mediators involved in promoting inflammation^29^. Early epidemiology research focused on the COX pathways and investigated the role of ARA metabolism in cancer^30^, and there are fewer reports on the role of LOX pathway in colon polyps and colon cancer. Among the LOX pathways, 5-LOX is closely associated with inflammation and carcinogenesis^29^, and 5-LOX expression is associated with colon adenomas and increased polyp size^31^. Overexpression of 5-LOX has been found in human cancers at different sites including prostate, pancreatic, colon, bladder, esophageal, and testicular cancer^32–35^. 5-LOX products formed by these cancer cells or other tumor associated cells can upregulate and contribute to tumor growth^36^. Here we report elevated plasma 5-HETE in patients with at least one or more colon polyps, and in patients with hyperplastic polyps or adenomas. These results are in agreement with the findings from a murine and human studies which reported 5-LOX is up-regulated in colon polyps compared to normal colonic mucosa^31,37^. In our study, the association between elevated 5-HETE and colon polyp type remained significant even after adjustment of BMI and WC. Individuals with elevated 5-HETE concentrations were at least six times more likely to have colon polyps compared to patients with no polyps. Moreover, other ARA-derived oxylipids synthesized through LOX pathways were not significantly associated with any responses from our study. Taken together, our observation may indicate that plasma 5-HETE concentrations could be an early indicator of malignant progression.

11-HETE is an ARA-derived oxylipid synthesized *de novo* through non-enzymatic oxidation and has been described as a marker of lipid peroxidation^38^. 11-HETE can also be produced enzymatically as a by-product of prostaglandin biosynthesis via the COX pathway^39^. Previous studies indicate increased serum 11-HETE is associated with diseases ranging from coronary events to cancers^20^. To our knowledge, we are the first group to report an association between elevated 11-HETE concentrations and colon polyps. The elevated levels of 11-HETE were observed in patients with hyperplastic polyps and adenomas. One possible explanation is that 11-HETE, generated from enzymatic oxidations through COX pathways, could have some function involved in tissue microenvironment that favors tumor cell growth, proliferation, and metastases^29^. On the other hand, since 11-HETE has also been described as a marker of lipid peroxidation, increased plasma 11-HETE could also be indicative of elevated oxidative stress and increased reactive oxygen species (ROS)^38^. Unfortunately our method did not employ a chiral HPLC column so we were unable to further speculate the potential source of 11-HETE production, however, it is possible that the source 11-HETE (i.e., COX-2 or ROS) could be determined by assessing concentration of 11-HETE enantiomers^40^. Future studies should verify that 11-HETE is associated with colon polyp presence and type in larger more diverse population, and determine the stereochemistry of 11-HETE to determine if elevated levels are due to biosynthesis through enzymatic, non-enzymatic, or a combination of both reactions in order better understand the association between 11-HETE and colon polyps.

Interestingly, our group previously reported concentrations of non-esterified 5-HETE and 11-HETE were positively associated with increased BMI and WC, in the same study population as our current study^20^. Our group also previously reported in these study participants that increased BMI was associated with an increased likelihood of adenoma presence compared to those with no polyps^6^. In our current study, we performed two statistical models that were adjusted for BMI and WC, and observed 5- and 11-HETE remained significantly associated with polyp type. Therefore, we speculate it is possible that increased levels of 5- and 11-HETE may serve as indicators of colon polyps independent of obesity in Caucasian males. One limitation of our study is that patients’ dietary patterns were not collected at the time of their colonoscopy. Previous epidemiologic studies suggest that high-fat diets are associated with increased incidence of tumors at different organ sites including colon^41,42^. Therefore, it is not possible to calculate associations between PUFA dietary intake and colon polyp types in this study. However, our results were consistent even after normalizing oxylipid concentrations to their parent PUFA, to account for the potential effects of altered levels of PUFA substrates (e.g., through dietary intake). Recently Butler *et al*. reported that PUFA percentage of total free plasma fatty acids were associated with colon cancer risk^43^. Since there is no longer plasma remaining from our study subjects for a similar follow-up lipid analysis, we were unable to normalize oxylipids to precursor PUFA percentage of total free plasma fatty acids. Recently, Haid *et al*. reported that plasma metabolite levels were altered after storage at −80 °C for 5 years^44^, and the oxylipid analysis of our patient samples were conducted 5 years after collection. While degradation across our patient samples would have been consistent since fresh unthawed plasma was used in our analysis, we recognize reported concentrations of oxylipids in our study may be lower than concentrations reported in plasma stored for a shorter period of time. This is an important consideration for follow-up studies targeting the tertile concentration cutoffs reported in our study.

## Conclusion

In conclusion, we report specific ARA-derived oxylipids produced through distinct oxygenating pathways are highly associated with colon polyp type. The oxylipids 5- and 11-HETE were significantly associated with polyps in all statistical models, even after adjusting for false discoveries and accounting for known risk factors such as age, smoking, and either BMI and WC. Future studies could be carried out in a large cohort to see if these oxylipids levels could be used as potential diagnostic measures for colon polyps, or if they can serve as independent predictors for colon polyp risks.

## References

[1] Colucci PM, Yale SH, Rall CJ (2003). Colorectal polyps. Clin Med Res.

[2] Kelly JK, Gabos S (1987). The pathogenesis of inflammatory polyps. Dis Colon Rectum.

[3] Siegel RL, Miller KD, Jemal A (2017). Colorectal Cancer Mortality Rates in Adults Aged 20 to 54 Years in the United States, 1970–2014. JAMA.

[4] Siegel RL (2017). Colorectal Cancer Incidence Patterns in the United States, 1974–2013. J Natl Cancer Inst.

[5] Wernli KJ (2010). Body size, IGF and growth hormone polymorphisms, and colorectal adenomas and hyperplastic polyps. Growth Horm IGF Res.

[6] Comstock SS (2014). Adipokines and obesity are associated with colorectal polyps in adult males: a cross-sectional study. PLoS One.

[7] Pickens CA (2015). Plasma phospholipids, non-esterified plasma polyunsaturated fatty acids and oxylipids are associated with BMI. Prostaglandins Leukot Essent Fatty Acids.

[8] Pickens, C.A., Albuquerque Pereira, M. F. & Fenton, J. I. Long-chain omega-6 plasma phospholipid polyunsaturated fatty acids and association with colon adenomas in adult men: a cross-sectional study. *Eur J Cancer Prev*. 2016. 10.1097/CEJ.0000000000000312 27768609

[9] Mattmiller SA (2014). Reduced macrophage selenoprotein expression alters oxidized lipid metabolite biosynthesis from arachidonic and linoleic acid. J Nutr Biochem.

[10] Arnold C (2010). Arachidonic acid-metabolizing cytochrome P450 enzymes are targets of {omega}-3 fatty acids. J Biol Chem.

[11] Smith WL, DeWitt DL, Garavito RM (2000). Cyclooxygenases: structural, cellular, and molecular biology. Annu Rev Biochem.

[12] Mabalirajan, U., Agrawal, A. & Ghosh, B. *15-Lipoxygenase eicosanoids are the putative ligands for vanilloid receptors and peroxisome proliferator-activated receptors (PPARs)*. *Proc Natl Acad Sci USA*, **109**(1), p. E1; author reply E2 (2012). 10.1073/pnas.1118477109 PMC3252933 22207620

[13] Mavangira, V. & Sordillo, L. M. Role of lipid mediators in the regulation of oxidative stress and inflammatory responses in dairy cattle. *Res Vet Sci*. (2017). 10.1016/j.rvsc.2017.08.002 28807478

[14] Bamia C (2013). Mediterranean diet and colorectal cancer risk: results from a European cohort. Eur J Epidemiol.

[15] Igal RA (2010). Stearoyl-CoA desaturase-1: a novel key player in the mechanisms of cell proliferation, programmed cell death and transformation to cancer. Carcinogenesis.

[16] Liu X, Strable MS, Ntambi JM (2011). Stearoyl CoA desaturase 1: role in cellular inflammation and stress. Adv Nutr.

[17] Murff HJ (2012). Dietary intake of PUFAs and colorectal polyp risk. Am J Clin Nutr.

[18] Murff HJ (2009). A prospective study of dietary polyunsaturated fatty acids and colorectal cancer risk in Chinese women. Cancer Epidemiol Biomarkers Prev.

[19] Kim J (2017). Association between dietary fat intake and colorectal adenoma in korean adults A cross-sectional study. Medicine.

[20] Pickens CA (2017). Obesity is positively associated with arachidonic acid-derived 5- and 11-hydroxyeicosatetraenoic acid (HETE). Metabolism.

[21] Comstock SS (2014). Cross-sectional analysis of obesity and serum analytes in males identifies sRAGE as a novel biomarker inversely associated with diverticulosis. PLoS One.

[22] Comstock SS (2014). Association of insulin-related serum factors with colorectal polyp number and type in adult males. Cancer Epidemiol Biomarkers Prev.

[23] Pickens, C. *et al*. Obesity is Associated with Changes in Plasma Oxylipids. *Faseb Journal*, **29** (2015).

[24] Pickens CA (2016). Altered Saturated and Monounsaturated Plasma Phospholipid Fatty Acid Profiles in Adult Males with Colon Adenomas. Cancer Epidemiol Biomarkers Prev.

[25] Mavangira V (2015). Polyunsaturated fatty acids influence differential biosynthesis of oxylipids and other lipid mediators during bovine coliform mastitis. J Dairy Sci.

[26] Benjamini Y, Hochberg Y (1995). Controlling the False Discovery Rate - a Practical and Powerful Approach to Multiple Testing. Journal of the Royal Statistical Society Series B-Methodological.

[27] Bland JM, Altman DG (1995). Multiple Significance Tests - the Bonferroni Method. 10. British Medical Journal.

[28] Team, R. D. C., R: A Language and Environment for Statistical Computing, in the R Foundation for Statistical Computing, A. Vienna, Editor. (2011).

[29] Janakiram NB, Rao CV (2014). The role of inflammation in colon cancer. Adv Exp Med Biol.

[30] Levy GN (1997). Prostaglandin H synthases, nonsteroidal anti-inflammatory drugs, and colon cancer. FASEB J.

[31] Wasilewicz MP (2010). Overexpression of 5-lipoxygenase in sporadic colonic adenomas and a possible new aspect of colon carcinogenesis. Int J Colorectal Dis.

[32] Hennig R (2002). 5-Lipoxygenase and leukotriene B(4) receptor are expressed in human pancreatic cancers but not in pancreatic ducts in normal tissue. Am J Pathol.

[33] Ohd JF (2003). Expression of the leukotriene D4 receptor CysLT1, COX-2, and other cell survival factors in colorectal adenocarcinomas. Gastroenterology.

[34] Yoshimura R (2004). Relationship between lipoxygenase and human testicular cancer. Int J Mol Med.

[35] Chen X (2004). Overexpression of 5-lipoxygenase in rat and human esophageal adenocarcinoma and inhibitory effects of zileuton and celecoxib on carcinogenesis. Clin Cancer Res.

[36] Radmark O (2015). 5-Lipoxygenase, a key enzyme for leukotriene biosynthesis in health and disease. Biochim Biophys Acta.

[37] Melstrom LG (2008). Overexpression of 5-lipoxygenase in colon polyps and cancer and the effect of 5-LOX inhibitors in vitro and in a murine model. Clin Cancer Res.

[38] Guido DM, McKenna R, Mathews WR (1993). Quantitation of hydroperoxy-eicosatetraenoic acids and hydroxy-eicosatetraenoic acids as indicators of lipid peroxidation using gas chromatography-mass spectrometry. Anal Biochem.

[39] Xiao G (1997). Analysis of hydroperoxide-induced tyrosyl radicals and lipoxygenase activity in aspirin-treated human prostaglandin H synthase-2. Biochemistry.

[40] Kuhn H (1987). Analysis of the stereochemistry of lipoxygenase-derived hydroxypolenoic fatty acids by means of chiral phase high-pressure liquid chromatography. Analytical Biochemistry.

[41] Rose DP (1997). Effects of dietary fatty acids on breast and prostate cancers: evidence from in vitro experiments and animal studies. Am J Clin Nutr.

[42] Rose DP (1997). Dietary Fat, Fatty Acids and Breast Cancer. Breast Cancer.

[43] Butler LM (2017). Plasma fatty acids and risk of colon and rectal cancers in the Singapore Chinese Health Study. NPJ Precis. Oncol.

[44] Haid M (2018). Long-Term Stability of Human Plasma Metabolites during Storage at −80 degrees C. J Proteome Res.

